# Species- and genome-wide dissection of the shoot ionome in *Brassica napus* and its relationship to seedling development

**DOI:** 10.3389/fpls.2014.00485

**Published:** 2014-09-30

**Authors:** Anja Bus, Niklas Körber, Isobel A. P. Parkin, Birgit Samans, Rod J. Snowdon, Jinquan Li, Benjamin Stich

**Affiliations:** ^1^Quantitative Crop Genetics, Max Planck Institute for Plant Breeding ResearchCologne, Germany; ^2^Crop Genetics and Biotechnology Unit, Institute of Crop Science and Resource Conservation, University of BonnBonn, Germany; ^3^Saskatoon Research Centre, Agriculture and Agri-Food CanadaSaskatoon, SK, Canada; ^4^Department of Plant Breeding, Research Centre for Biosystems, Land Use and Nutrition, Justus Liebig UniversityGiessen, Germany

**Keywords:** genome-wide association mapping, single nucleotide polymorphism, shoot ionome, *Brassica napus*, linkage disequilibrium

## Abstract

Knowing the genetic basis of the plant ionome is essential for understanding the control of nutrient transport and accumulation. The aim of this research was to (i) study mineral nutrient concentrations in a large and diverse set of *Brassica napus*, (ii) describe the relationships between the shoot ionome and seedling development, and (iii) identify genetic regions associated with variation of the shoot ionome. The plant material under study was a germplasm set consisting of 509 inbred lines that was genotyped by a 6K single nucleotide polymorphism (SNP) array and phenotyped by analyzing the concentrations of eleven mineral nutrients in the shoots of 30 days old seedlings. Among mineral concentrations, positive correlations were found, whereas mineral concentrations were mainly negatively correlated with seedling development traits from earlier studies. In a genome-wide association mapping approach, altogether 29 significantly associated loci were identified across seven traits after correcting for multiple testing. The associations included a locus with effects on the concentrations of Cu, Mn, and Zn on chromosome C3, and a genetic region with multiple associations for Na concentration on chromosome A9. This region was situated within an association hotspot close to *SOS1*, a key gene for Na tolerance in plants.

## 1. Introduction

Plant cells depend on the presence of nutritional elements, which fulfill a variety of functions, where elements act, amongst others, as regulators, cofactors, or structural components (Baxter, 2009). The composition of plant mineral nutrients and trace elements is referred to as the ionome (Salt et al., 2008). Its studies, so-called ionomics, aim to reveal knowledge about these functions and the networks controlling uptake, transport, and accumulation of elements. Ionomics have become of great interest in research due to its relevance in plant development and performance, where each nutrient plays a specific role. However, some elements have similar chemical properties and therefore compete for uptake or carriers (Marschner, 1995). Ionomics approaches therefore do not only address the question of quantifying nutrient concentrations but also how ions interact with each other, which factors influence their uptake and accumulation, and how the ionome can serve as indicator for associated traits.

Previous studies have characterized either partly, or as a whole, the plant ionome and used this knowledge to gain further insights into the genetic or physiologic architecture. Lahner et al. (2003) quantified 18 elements in shoots of 6000 mutagenized plants of *Arabidopsis thaliana* and analyzed the mutation frequency based on the elemental profiles. Their findings led to the conclusion that about 2–4% of the genome is involved in the regulation of the nutrient and trace element content in the species. Another extensive study of the ionome of *A. thaliana* under different iron and phosphorus conditions showed through logistic regression modeling that there is a relationship between the ionome and specific physiological responses, e.g., to Fe or P deficiency (Baxter et al., 2008). Although most of the knowledge comes from model species, ionomics approaches have recently also been performed in crops such as maize (Baxter et al., 2013), soybean (Ziegler et al., 2013), and barley (Wu et al., 2013). For the genus *Brassica* there is limited knowledge on the genetic basis of ionomic variation. A QTL analysis on loci controlling mineral concentrations in *B. napus* under normal and deficient boron conditions (Liu et al., 2009) revealed specific correlations among seven different elements and several QTLs as well as epistatic interaction pairs for mineral concentrations. These were specific for each boron regime, implying that mineral homoeostasis is controlled by genetic factors and that ion transport under different regimes is driven by multiple genes. In *B. rapa*, the species that forms the *B. napus* A genome, ten QTLs were detected for concentrations of 11 minerals under different Zn regimes (Wu et al., 2008). In *B. oleracea*, which in turn represents the C genome of *B. napus*, there are a number of known QTLs for the highly heritable Ca and Mg concentrations in shoots, of which the most significant QTLs are localized on chromosome C9 (Broadley et al., 2008). Moreover, it is known from a study on *B. napus* seeds that, on the one hand, multiple loci control mineral concentrations and, on the other hand, physiological and molecular mechanisms driving mineral accumulation are likely to be shared between elements (Ding et al., 2010). However, no study has provided a species- and genome-wide analysis of the *B. napus* ionome.

A healthy and robust development of the seedling is the prerequisite for a vigorous, high-yielding crop. Understanding the genetic basis of the shoot ionome of young plants will give deeper insights into how nutrient transport and accumulation are controlled. Furthermore, studying the relationship between ionomics and seedling development will allow an understanding of so far unknown interactions.

With regard to the aforementioned studies, association mapping (AM) based on linkage disequilibrium (LD) is a complementary approach for the detection of associations between quantitative traits and variation on the molecular level in crops. AM allows to discover the genetic bases for traits under certain scenarios, including when contributing alleles are at moderate frequencies within the population (Oraguzie et al., 2007). The decreasing costs of single nucleotide polymorphism (SNP) arrays and the advent of next-generation sequencing technologies allow the development of thousands of SNPs even in the allotetraploid *B. napus* (Trick et al., 2009; Bus et al., 2012; Snowdon and Iniguez Luy, 2012; Edwards et al., 2013). Therefore, SNPs have become the marker type of choice for genome-wide AM. As whole-genome resequencing is still laborious and expensive, mostly transcriptomics techniques have been chosen as high-throughput SNP detection method for *B. napus*. These approaches have been carried out on relatively small SNP and plant sets. Here we present an AM study on a large-size worldwide germplasm set that extensively represents the species *B. napus*, using a 6K SNP array to allow high resolution mapping. The objectives of our study were to (i) examine mineral nutrient concentrations and their interactions in a large and diverse set of *B. napus*, (ii) study the relationships between the shoot ionome and seedling development traits, and (iii) identify genetic regions associated with variation of the shoot ionome.

## 2. Materials and methods

We investigated a species-wide diversity set of *B. napus*, consisting of 509 inbreds, of which the genetic diversity and population structure had been characterized with 89 simple sequence repeat (SSR) markers as described by Bus et al. (2011). The diversity set consists of 183 winter oilseed rape (OSR), 22 winter fodder, 73 swede (*B. napus* ssp. *napobrassica*), 7 semi-winter OSR, 204 spring OSR, 4 spring fodder, 10 vegetable, and 6 unspecified inbred lines.

For ionome and seedling development analysis, the plant material was grown in a greenhouse experiment that comprised six replicates which were performed in a row within a period of 6 months, where each replicate lasted 30 days (Körber et al., 2012). The experimental design was an alpha lattice with 24 blocks, each of 24 pots. The temperature was set to 24°C during 16 h of light exposure and to 18°C during 8 h of dark exposure. Seeds were sown in 10 × 10 cm pots filled with soil (Einheitserde, Balster Einheitserdewerk, Fröndenberg, Germany). During cultivation, the plants were treated twice with liquid fertilizer Wuxal Super 8-8-6 (Haug, Ammerbuch, Germany). From 8 to 16 days after sowing, digital infrared images of the seedlings were taken. A mask of each plant was created using a color threshold, and the projected leaf areas (LA08–LA16) of the seedlings were calculated using the digital image processing software ImageJ 1.42q (http://rsb.info.nih.gov/ij/) (Table 1). Other seedling development traits were derived from the plant masks at 10 days after sowing (Table 1). Growth parameters *a* (intercept, PRA) and *k* (growth factor, PRK) were calculated from LA08–LA16 by non-linear regression. Furthermore, data on the relative leaf chlorophyll content as measured by Minolta company-defined SPAD (Soil Plant Analysis Development) values (SPD), fresh mass (FHM), dry mass (DYM), and H_2_O content (H2O) of the plants were collected after a growing period of 30 days after sowing. Plant material for ionome analysis was harvested from 30 days old seedlings, oven-dried, and lyophilized.

**Table 1:** **Abbreviations of eleven mineral concentrations and twenty seedling development traits, broad sense heritability on an entry mean basis (*h*^2^), and the percentage of phenotypic variation explained by population structure (*r*^2^) measured in a *B. napus* germplasm set**.

**Abbreviation**	**Trait**	**Unit**	***h*^2^**	***r*^2^**
**MINERAL CONCENTRATIONS**				
B	Shoot boron concentration	μg/g	0.74	1.57
Ca	Shoot calcium concentration	mg/g	0.85	4.54
Cu	Shoot copper concentration	μg/g	0.82	1.02
Fe	Shoot iron concentration	μg/g	0.46	0.05
K	Shoot potassium concentration	mg/g	0.77	0.15
Mg	Shoot magnesium concentration	mg/g	0.77	0.88
Mn	Shoot manganese concentration	μg/g	0.80	0.03
Na	Shoot sodium concentration	mg/g	0.83	5.46
P	Shoot phosphorus concentration	mg/g	0.43	0.01
S	Shoot sulfur concentration	mg/g	0.86	1.40
Zn	Shoot zinc concentration	μg/g	0.82	0.90
**SEEDLING DEVELOPMENT TRAITS**				
LA08	Projected leaf area at day 8	cm^2^	0.82	5.89
LA10	Projected leaf area at day 10	cm^2^	0.84	5.39
LA12	Projected leaf area at day 12	cm^2^	0.81	5.77
LA14	Projected leaf area at day 14	cm^2^	0.76	5.30
LA16	Projected leaf area at day 16	cm^2^	0.73	4.86
PRA	Parameter *a*	cm^2^	0.51	2.93
PRK	Parameter *k*	1/day	0.28	0.12
PER	Plant perimeter length	cm	0.80	4.43
MAJ	Plant major axis of the best fitting ellipse	cm	0.82	3.92
MIN	Plant minor axis of the best fitting ellipse	cm	0.78	5.22
ASR	Plant aspect ratio: major axis/minor axis		0.56	0.04
MAD	Maximum plant diameter	cm	0.82	3.94
MID	Minimum plant diameter	cm	0.81	4.64
CIR	Plant circularity: 4π(area/perimeter^2^)		0.33	0.49
ROU	Plant roundness: 4 × area/(π × major axis^2^)		0.52	0.00
SOY	Plant solidity: area/convex area		0.52	0.02
FHM	Fresh mass	g	0.69	2.02
DYM	Dry mass	g	0.72	4.00
SPD	SPAD measurement		0.77	3.59
H2O	H_2_O content	% of fresh mass	0.39	6.67

The entirety of all investigated mineral concentrations is herein referred to as the shoot ionome. The concentrations of altogether eleven plant nutritional elements, B, Ca, Cu, Fe, K, Mg, Mn, Na, P, S, and Zn (Table 1), were analyzed by inductively coupled plasma optical emission spectrometry (ICP-OES, Vista Pro Radial, Varian, Palo Alto, CA, USA) at Landesanstalt für Landwirtschaftliche Chemie at the University of Hohenheim (Stuttgart, Germany).

Plant genomic DNA for SNP genotyping was extracted from fresh or lyophilized leaf material using the BioSprint 96 DNA Plant Kit and the BioSprint 96 robotic workstation (Qiagen), following the manufacturer's protocol. Genotyping of the SNPs was performed at Agriculture and Agri-Food Canada using a custom Illumina Infinium 6K array (http://aafc-aac.usask.ca/ASSYST/). Only SNPs with a minor allele frequency larger than 5% were included in the association analysis. Of all of the SNPs from the array, altogether 3910 SNPs were used, and SNP data were available for 505 inbreds of the germplasm set.

### 2.1. Statistical analyses

Adjusted entry means (AEM) were calculated for each genotype-mineral concentration combination using the following mixed model:

(1) $$y_{ikm}=\mu +g_{i}+r_{k}+b_{km}+e_{ikm},$$

where *y_ikm_* was the observation of the *i*th genotype in the *m*th block of the *k*th replicate, μ the general mean, *g_i_* the effect of the *i*th genotype, *r_k_* the effect of the *k*th replicate, *b_km_* the effect of the *m*th block in the *k*th replicate, and *e_ikm_* the residual error. Outliers in residual plots were discarded. For the calculation of AEM, we considered *g* as fixed and all other effects as random. For estimating the genotypic variance (σ^2^_*g*_) and the error variance (σ^2^_*e*_), all effects were considered as random. The heritabilities *h*^2^ of each mineral concentration on an entry mean basis were calculated as follows:

(2) $$h^{2}=\frac{\sigma_{g}^{2}}{\sigma_{g}^{2}+\frac{\sigma_{e}^{2}}{b}},$$

where *b* was the number of replicates. Variance components and AEM were determined by the restricted maximum likelihood (REML) method. The mixed model analyses were performed with ASREML release 2.0 (Gilmour et al., 2006).

The Pearson partial correlation coefficient (Fisher, 1924) was calculated for all pairs of traits, with significance levels corrected according to Holm's method (Holm, 1979). Associations among mineral concentrations as well as between mineral concentrations and the seedling development traits described by Körber et al. (2012) were visualized by principal component analysis (PCA). To do so, phenotype data were processed by using the R prcomp() function. All of the 509 genotypes of this study had been assigned to three clusters based on SSR marker data (Bus et al., 2011). Whenever population structure needed to be accounted for in our statistical analyses, the germplasm set was separated into these clusters, referred to as MCLUST clusters 1, 2, and 3. Multiple stepwise regression of seedling development traits was run on mineral concentrations. To identify the optimum linear model which predicts the best representation of the seedling development traits, mineral concentrations were used as independent variables. The latter were selected based on the Bayesian information criterion (BIC) (Schwarz, 1978):

(3) $$y_{i}=\mu +\sum_{p\;=\;1}^{v}b_{p}x_{pi}+e_{i},$$

where *y_i_* was the observation of the *i*th genotype, for which the best set of predictors (mineral concentrations) was to be identified, μ the intercept term, *v* the number of selected variables, *b_p_* the regression coefficient of the *p*th mineral nutrient, *x_pi_* the AEM of the *p*th mineral nutrient for the *i*th genotype, and *e_i_* the residual.

### 2.2. Association analysis and assessment of linkage disequilibrium

The mixed model according to the PK method (Stich et al., 2008) was used to analyze associations between polymorphic sites and the mineral concentrations: The PK-mixed model was

(4) $$M_{ip}=\mu +a_{p}+\sum_{u\;=\;1}^{z}D_{iu}v_{u}+g_{i}^{*}+e_{ip},$$

where *M_ip_* was the entry mean of the *i*th entry carrying allele *p*, *a_p_* the effect of allele *p*, *v_u_* the effect of the *u*th column of the population structure matrix *D*, g^*^_i_ the residual genetic effect of the *i*th entry, and *e_ip_* the residual (Yu et al., 2006; Stich et al., 2008). The first and second principal coordinates from the SSR-based principal coordinate analysis in Bus et al. (2011) were used as *D* matrix for the association analysis. The kinship coefficient *K_ij_* between inbreds *i* and *j* were calculated according to Bernardo (1993). For the series of *T* values (describing the average probability that a variant from one parent of inbred *i* and a variant from one parent of inbred *j* are alike in state, given that they are not identical by descent) 0, 0.025, …, 0.975, K matrices between all inbreds were calculated. Negative kinship values between inbreds were set to 0. The optimum *T* value (showing the minimum likelihood) was calculated according to Stich et al. (2008). All statistical analyses, if not stated otherwise, were performed with statistical software R (R Development Core Team, 2011). For assuming an association, an adjusted *P* value (Bonferroni correction) of less than 0.05 was required. The association analysis of the mineral concentrations with the polymorphisms was carried out using the R package EMMA (Kang et al., 2008). The percentage of phenotypic variation explained by the significant SNPs was calculated according to Magee (1990). The threshold for significant LD (Hill and Robertson, 1968) was chosen to be a squared correlation of allele frequencies higher than 0.8. Linked SNPs in significant LD were assigned to genetic regions associated with the traits under study. Linked loci were defined as loci on the same chromosome and unlinked loci were defined as loci on different chromosomes.

### 2.3. Analysis of genes linked to associated SNPs

The 6K SNP array positions were derived from the reference genome sequences of *B. rapa* (Wang et al., 2011) and *B. oleracea* (Yu et al., 2013). To define the positions in the *B. napus* genome, BLAST searches (Altschul et al., 1990) with flanking regions adjacent to the SNPs were done against a pre-publication draft (version 4) of the *B. napus* “Darmor-Bzh” reference genome sequence assembly, which was kindly made available prior to public release by INRA, France, Unité de Recherche en Génomique Végétale (Boulos Chaloub, INRA-URGV, Evry, France, unpublished data). For each SNP, the BLAST hit with the highest score was selected. If the two highest scores were identical or very similar, both positions were selected. SNPs that could not be assigned to a *B. napus* chromosome were excluded from further searches. For each locus an interval of 700 kb upstream and downstream of the defined position was screened for annotated genes. Predicted genes' sequences were annotated using Blast2GO (Conesa et al., 2005). A Gene Ontology (GO) enrichment analysis was performed using the bioconductor package GOstat (Beissbarth and Speed, 2004). The enriched GO terms of each trait and across all traits were mapped to plant GO slim terms using GOSlimViewer (McCarthy et al., 2006).

## 3. Results

**Figure 1:**
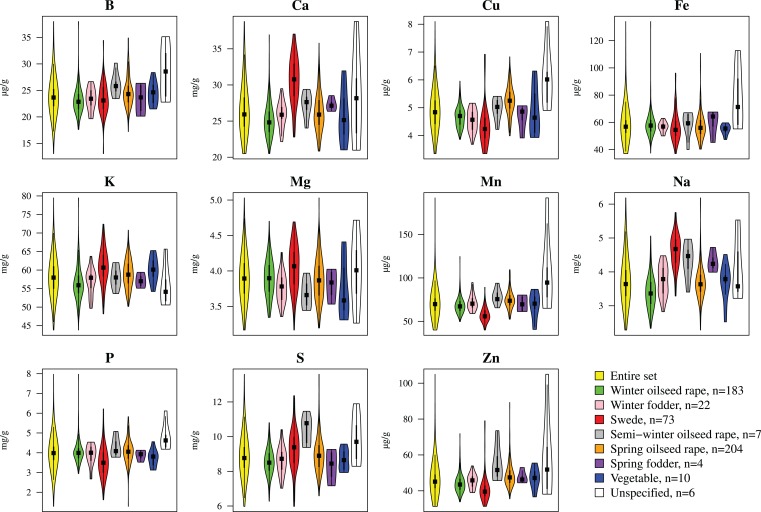
**Frequency distribution of adjusted entry means determined for eleven different mineral concentrations in a germplasm set of *B. napus* inbreds**. Colors represent different germplasm types. Yellow plots represent the entire germplasm set of 509 inbreds. The number of genotypes for each germplasm type is given in the legend. In each plot, a marker denotes the median of the data, a box indicates the interquartile range, and spikes extend to the upper and lower adjacent values, overlaid is the density.

**Figure 2:**
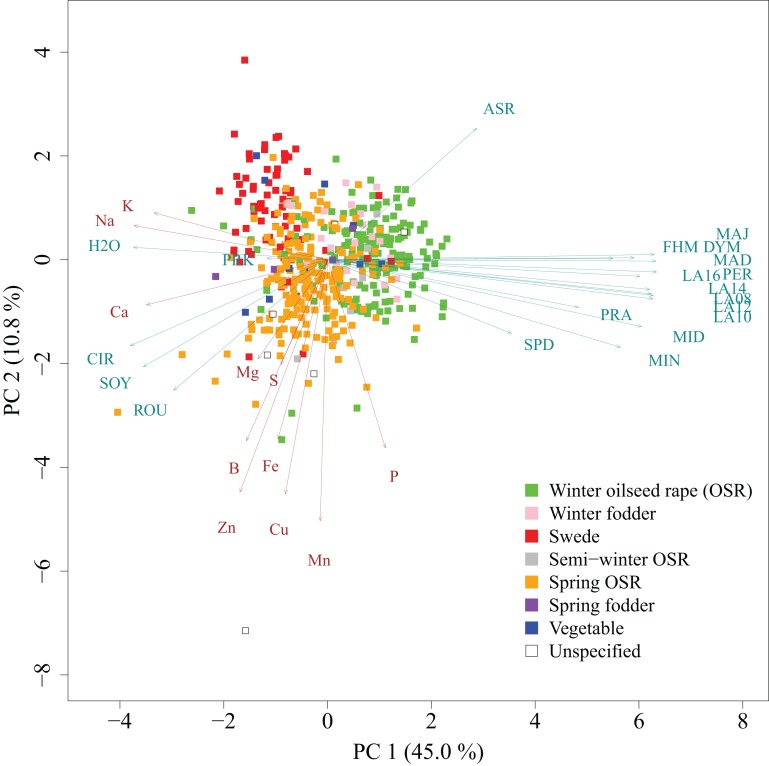
**Principal component analysis of 509 *B. napus* inbreds (points), seedling development traits (turquoise arrows), and mineral concentrations (brown arrows)**. Germplasm types are represented by different colors. PC 1 and PC 2 are the first and second principal components, respectively. The proportion of variance explained by the principal components is given in parentheses.

We determined the mineral concentrations of eleven elements in a germplasm set of 509 inbred lines. The AEM of mineral concentrations were approximately normally distributed (Figure 1). Heritabilities *h*^2^ of mineral concentrations represented a range of 0.43 (P) to 0.86 (S) with a mean of 0.74 (Table 1). The phenotypic variation explained by population structure *r*^2^ ranged from 0.01 (P) to 5.46% (Na), with a mean of 1.46% (Table 1). In the PCA representing the 509 *B. napus* inbreds as well as the mineral concentrations and the seedling development traits, the first two principal components explained 45.0 and 10.8% of the variance (Figure 2). With respect to these two principal components, all inbreds of the germplasm types winter OSR, winter fodder, semi-winter OSR, spring OSR, and swedes were assigned to overlapping clusters, whereas spring fodder, vegetable, and unspecified lines did not show specific clustering patterns. The seedling development traits H2O, PRK, MAJ, FHM, DYM, MAD, PER, LA16, LA14, LA08, LA12, LA10, PRA, MID, MIN, and SPD and the concentrations of elements K, Na, and Ca had loadings mainly on PC 1, whereas Mn, Cu, Fe, Zn, S, B, and P primarily showed loadings on PC 2. ASR, CIR, SOY, ROU, and Mg contributed to both PC 1 and PC 2.

**Figure 3:**
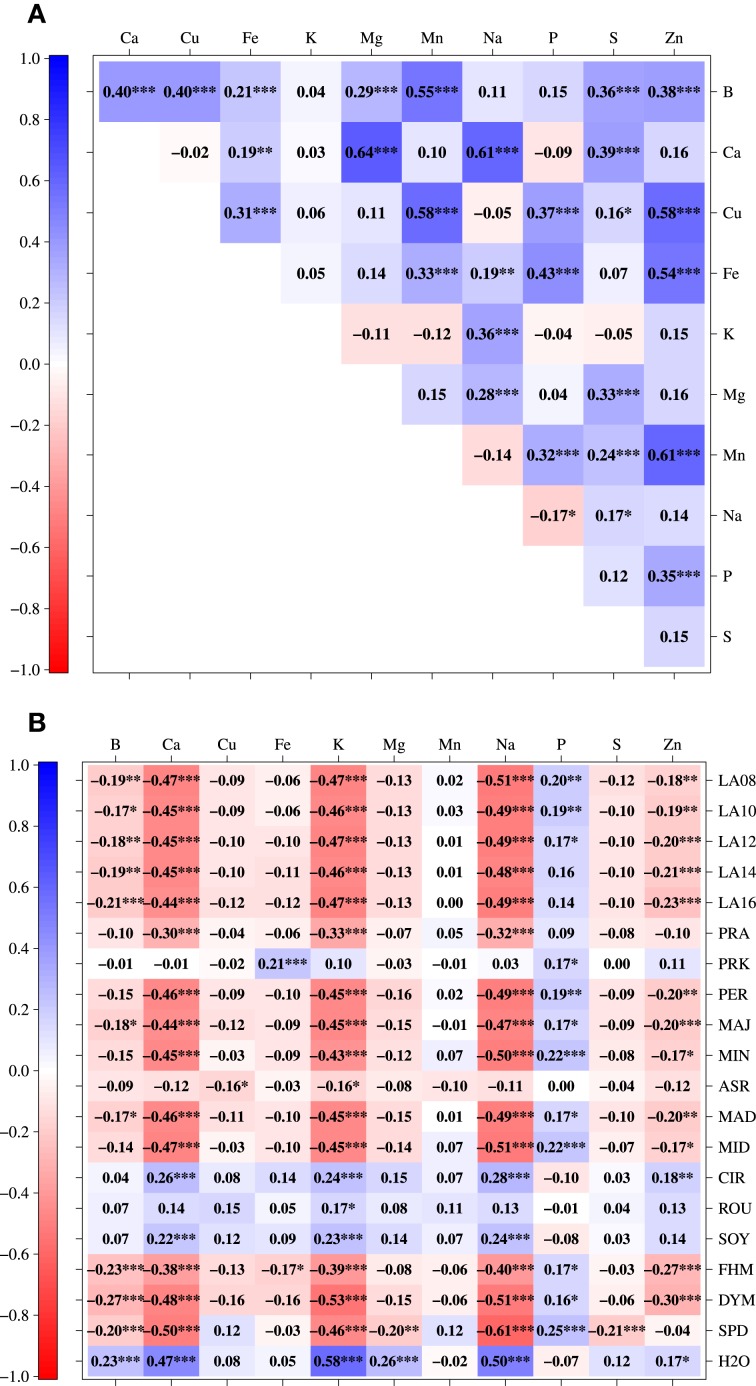
Pearson correlation coefficients (^*^P ≤ 0.05, ^**^P ≤ 0.01, ^***^P ≤ 0.001, corrected using Holm's method) between pairs of eleven different mineral concentrations in 509 *B. napus* inbreds (A) and between pairs of mineral concentrations and seedling development traits (B).

Patterns of correlations among mineral concentrations described strong relationships between B/Mn, Ca/Mg, Ca/Na, Cu/Mn, Cu/Zn, Fe/Zn, and Mn/Zn (Figure 3A), which were all positively correlated with each other. Between mineral concentrations and seedling development traits there were high negative correlations between each of Ca, K, and Na and all seedling development traits except PRK, ASR, CIR, ROU, SOY, and H2O. The latter, however, was tightly positively correlated with Ca, K, and Na (Figure 3B).

The proportions of the phenotypic variation (adjusted *R*^2^) of the seedling development traits that could be explained by a linear combination of a selected set of the mineral concentrations in the germplasm set ranged from 0.042 (PRK) to 0.570 (DYM) (Table 2). The mean adj. *R*^2^ observed for the entire set was 0.356.

**Table 2:** **Optimum linear combinations of eleven mineral concentrations describing seedling development traits in 509 *B. napus* inbreds, and the proportion of phenotypic variation explained by the selected independent variables (adjusted *R*^2^)**.

**Trait**	**Optimum linear model**	**Adjusted *R*^2^**
LA08	8.715 − 0.142 Ca − 0.212 Cu − 0.075 K + 0.511 Mg + 0.243 P	0.484
LA10	15.875 − 0.249 Ca − 0.392 Cu − 0.133 K + 0.872 Mg + 0.422 P	0.453
LA12	25.098 − 0.388 Ca − 0.598 Cu − 0.210 K + 1.417 Mg + 0.580 P	0.456
LA14	40.992 − 0.622 Ca − 0.954 Cu − 0.336 K + 2.174 Mg + 0.880 P	0.436
LA16	76.048 − 1.134 Ca − 1.949 Cu − 0.630 K + 4.069 Mg + 1.532 P	0.449
PRA	1.239 − 0.019 Ca − 0.011 K + 0.077 Mg	0.202
PRK	0.230 + 0.001 Fe	0.042
PER	32.008 + 0.097 B − 0.448 Ca − 0.799 Cu − 0.227 K + 1.208 Mg + 0.696 P	0.452
MAJ	7.853 − 0.096 Ca − 0.176 Cu − 0.053 K + 0.296 Mg + 0.163 P	0.426
MIN	3.636 − 0.045 Ca − 0.023 K + 0.164 Mg + 0.090 P − 0.005 Zn	0.424
ASR	2.550 − 0.007 Ca − 0.049 Cu − 0.007 K	0.058
MAD	8.521 − 0.109 Ca − 0.180 Cu − 0.058 K + 0.341 Mg + 0.172 P	0.441
MID	4.564 − 0.057 Ca − 0.030 K + 0.190 Mg + 0.107 P − 0.006 Zn	0.455
CIR	0.170 − 0.002 B + 0.004 Ca + 0.002 K − 0.009 P + 0.001 Zn	0.150
ROU	0.326 + 0.003 Ca + 0.015 Cu + 0.002 K	0.058
SOY	0.553 + 0.003 Ca + 0.006 Cu + 0.002 K	0.106
FHM	27.014 − 0.439 Ca − 0.596 Cu − 0.189 K + 1.956 Mg + 0.928 P + 0.302 S − 0.072 Zn	0.378
DYM	3.198 − 0.046 Ca − 0.064 Cu − 0.026 K + 0.134 Mg + 0.080 P + 0.028 S − 0.006 Zn	0.570
SPD	62.798 − 0.127 B − 0.233 Ca + 0.683 Cu − 0.269 K − 1.584 Na + 0.807 P − 0.328 S	0.537
H2O	84.330 + 0.099 Ca + 0.089 K	0.543

**Figure 4:**
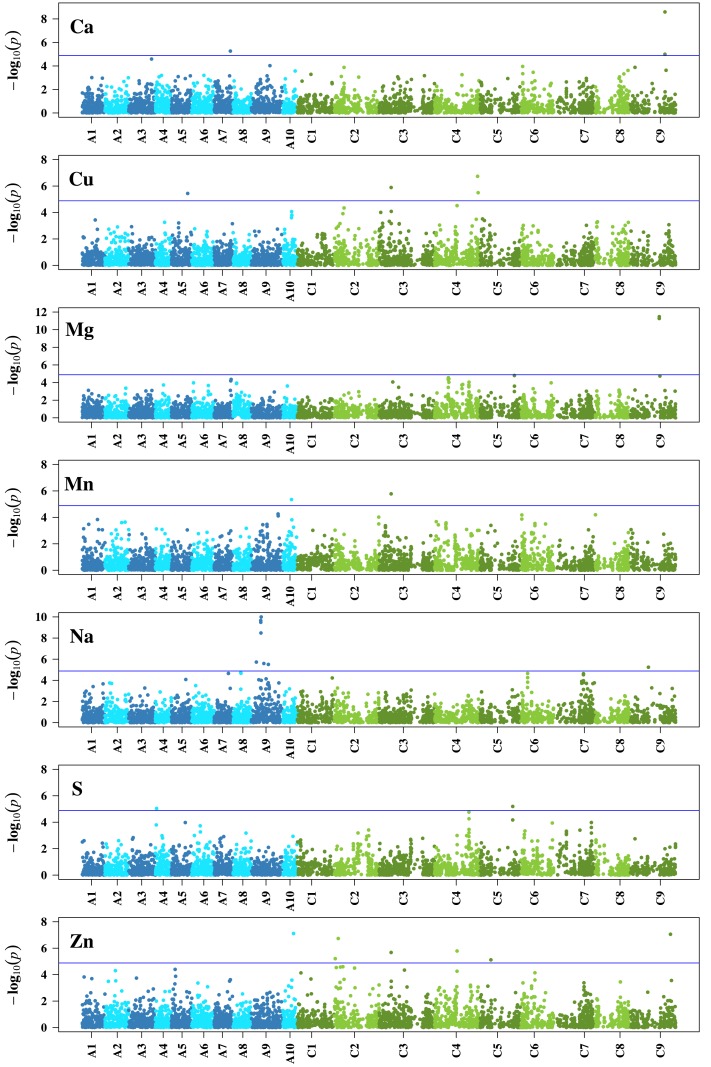
**Genome-wide *P*-values for association analysis of seven mineral concentrations for which significant (α = 0.05 prior to Bonferroni correction) associations were identified in a *B. napus* diversity set using a 6 K single nucleotide polymorphism array after correction for multiple testing**. Chromosomes of the *B. napus* A genome are colored blue, chromosomes of the *B. napus* C genome are colored green. The blue line shows the significance threshold after Bonferroni correction.

In the association analysis, we found altogether 29 significant (α = 0.05, Bonferroni correction) associations of 27 unique SNPs with Ca (3 associations), Cu (4 associations), Mg (2 associations), Mn (2 associations), Na (8 associations), S (2 associations), and Zn (8 associations) (Figure 4). One locus (Bn-ctg7180014703649-p1976) was associated with the three traits Cu, Mn, and Zn (Table 3). None of the SNPs was significantly associated with B, Fe, K, and P (Supplementary Figure S1). The phenotypic variation explained by single associated markers ranged from 3.02% (Bn-ctg7180014761247-p14206 and Cu) to 9.73% (p6_1705_snp15 and Mg), and the variation explained by all associated markers, determined in a simultaneous fit, ranged from 6.32% (S) to 22.89% (Zn) (Table 3).

**Table 3:** **Twenty-nine significant (α = 0.05) associations of 27 unique single nucleotide (SNP) markers with mineral concentrations after Bonferroni correction in a *B. napus* diversity set**.

**Trait**	**SNP array code**	**Genetic region**	**Chromosome**	**Position (bp)**	***P***	**Allele 1/2**	**Effect allele 1/2**	***P_V_***
Ca	Bn-ctg7180014762670-p15710	Ca-C9	C9	41581687	1.01E-05	T/G	−2.46	5.22
Ca	Bn-ctg7180014762670-p5913	Ca-C9	C9	41591486	2.54E-09	T/G	2.75	6.29
Ca	Bn-Scaffold000019-p2724372		A7	18986728	5.44E-06	A/G	1.12	3.09
Simultaneous fit								10.05
Cu	Bn-ctg7180014703649-p1976		C3	14030269	1.29E-06	T/C	0.38	4.90
Cu	Bn-ctg7180014761247-p14206		C4	51056931	3.19E-06	T/G	0.34	3.02
Cu	Bn-ctg7180014763826-p6024		C4	50413112	1.84E-07	A/G	0.33	5.13
Cu	Bn-Scaffold000037-p374106		A5	19096696	3.63E-06	A/G	0.29	4.72
Simultaneous fit								11.22
Mg	Bn-ctg7180014743366-p2908	Mg-C9	C9	34911341	5.40E-12	A/G	0.21	9.57
Mg	p6_1705_snp15	Mg-C9	C9	34961960	3.36E-12	A/G	0.21	9.73
Simultaneous fit								9.73
Mn	Bn-ctg7180014703649-p1976		C3	14030269	1.67E-06	T/C	7.63	3.86
Mn	Bn-Scaffold000002-p2620032		A10	10179686	4.49E-06	T/C	5.65	4.00
Simultaneous fit								6.59
Na	Bn-ctg7180014744730-p4896		C9	22227851	5.73E-06	T/C	−0.26	3.97
Na	Bn-Scaffold000022-p443574		A9	14774926	2.54E-06	T/G	0.25	3.59
Na	Bn-Scaffold000040-p1595095		A9	20042162	3.11E-06	A/G	0.28	4.06
Na	Bn-Scaffold000135-p101094	Na-A9	A9	11358358	3.32E-09	A/G	−0.37	7.71
Na	Bn-Scaffold000135-p393158	Na-A9	A9	11650422	1.02E-10	A/C	0.38	7.95
Na	Bn-Scaffold000145-p148088	Na-A9	A9	11109077	2.22E-10	A/G	0.37	7.71
Na	Bn-Scaffold000145-p66952	Na-A9	A9	11190213	3.24E-10	A/G	0.37	7.63
Na	Bn-Scaffold000481-p8079		A9	5977526	1.86E-06	A/G	0.27	4.41
Simultaneous fit								13.03
S	Bn-ctg7180014733138-p6820		C5	38046495	6.42E-06	T/C	0.45	3.58
S	Bn-Scaffold000016-p1218862		A4	1432875	9.13E-06	T/C	0.52	3.30
Simultaneous fit								6.32
Zn	Bn-ctg7180014703649-p1976		C3	14030269	2.10E-06	T/C	4.44	4.05
Zn	Bn-ctg7180014733119-p5210	Zn-C2	C2	1416161	6.18E-06	T/C	5.22	4.64
Zn	Bn-ctg7180014733119-p5336	Zn-C2	C2	1416035	6.18E-06	T/G	5.22	4.64
Zn	Bn-ctg7180014739774-p5570		C9	48038054	8.86E-08	T/G	7.09	6.31
Zn	Bn-ctg7180014753773-p3940		C2	4902638	1.86E-07	A/G	4.05	5.59
Zn	Bn-ctg7180014761298-p8042		C5	12500137	7.61E-06	T/G	4.94	3.81
Zn	Bn-ctg7180014776046-p1871		C4	26362515	1.63E-06	A/G	6.53	5.00
Zn	Bn-Scaffold000002-p221862		A10	12577856	7.76E-08	A/G	−5.72	6.46
Simultaneous fit								22.89

*Genetic region describes a common SNP region with linked loci that were associated with traits and in significant linkage disequilibrium with each other, *P* is the *P* value prior to Bonferroni correction, *P_V_* is the proportion of the explained phenotypic variation (%)*.

Linked SNPs from marker-trait associations that were in significant LD with each other were combined to represent genetic regions associated with the respective traits. For Ca, Mg, Na, and Zn, two (Ca, Mg, Zn) and four (Na) linked loci associated with the respective trait were in significant LD (Supplementary Table S1) and assigned to the genetic regions Ca-C9, Mg-C9, Na-A9, and Zn-C2 (Table 3). We therefore identified genetic regions on chromosomes A9, C2, and C9. None of the loci in significant LD were associated with different traits.

Based on the highest scores of the BLAST results, the chromosome information derived from the *B. rapa* and *B. oleracea* references was in accordance with the information derived from the *B. napus* reference assembly for 23 of all 27 unique SNPs with significant associations (Supplementary Table S2). In case there were two hits with the same or very similar top score, the information could be verified by one of them. We detected within the 700 kb interval each upstream and downstream the significant associations between 105 (Bn-Scaffold000002-p221862) and 524 (Bn-Scaffold000481-p8079) candidate genes. The GO enrichment analysis done on each trait revealed between 62 (Mg) and 207 (Na) enriched terms for the category biological process, between 6 (Ca, S) and 33 (Na) for cellular component, and between 27 (Mg) and 135 (Na) for molecular function (*P* ≤ 0.05) (Supplementary Table S3). Across all traits, the analysis resulted in 190 (biological process), 42 (cellular component), and 121 (molecular function) enriched terms. The enriched GO terms showed unequal representations of plant GO slim terms (Supplementary Figure S2). About 540 kb away from SNP Bn-Scaffold000022-p443574, which is located within the association hotspot for Na on chromosome A9 (Figure 4), we found the gene *SOS1* (Salt Overly Sensitive 1).

## 4. Discussion

### 4.1. Correlation between population structure and the shoot ionome

A serious issue with AM is the identification of spurious associations caused by the presence of population structure (Flint-Garcia et al., 2003). In the germplasm set under study, we had previously detected population structure, which became apparent through MCLUST clusters which were represented mainly by winter types, spring types, and swedes (Bus et al., 2011). The proportion of shoot ionome variation explained by population structure *r*^2^ was nevertheless low across the shoot ionome (Table 1), and mineral concentrations varied moderately across germplasm types (Figure 1), indicating that population structure had little influence on our phenotypic observations of the shoot ionome. Based on this information, we decided against a separate analysis for each MCLUST cluster.

### 4.2. Relationships between traits

We characterized the shoot ionome in a diverse panel of *B. napus*. The correlation coefficients among mineral concentrations were largely positive (Figure 3A). This observation suggests either common ion uptake and transport mechanisms shared between the respective elements or pleiotropy of the causal variants responsible for correlated mineral concentrations. The tightest positive correlation among mineral concentrations was found between Ca and Mg (0.64). The phenomenon of strong positive correlations between Ca and Mg accumulation in plants has been reviewed earlier (Baxter, 2009; White and Broadley, 2009). Similar to our results, Broadley et al. (2008) observed a high correlation (0.97) between the mean shoot Ca and Mg across ten *B. oleracea* subtaxa in an investigation of genetic variation of these two elements in 355 diverse accessions, 74 modern F_1_ cultivars, and a mapping population of *B. oleracea*. They pointed out that Ca and Mg homoeostasis is very likely to be controlled by common regulatory networks because they are chemically very similar. This is potentially also the reason for the tight correlation we found. The relationship between Ca and Mg in plants was furthermore described in a large study by Watanabe et al. (2007), who investigated 42 elements in more than 2000 leaf samples from 670 plant species and determined a correlation of 0.36 between Ca and Mg. The relationship between Ca and Mg we observed was therefore in line with previous studies.

Ding et al. (2010) measured the seed contents of Ca, Cu, Fe, Mg, Mn, P, and Zn in in 124 recombinant inbreds of *B. napus* under low and normal P conditions in two consecutive years. The correlations measured under the normal P regime across 2 years showed similar trends when compared with our data. However, major differences were found between Ca and P (−0.09 in our study vs. 0.49 in year 1 and 0.38 in year 2) as well as Mg and P (0.04 vs. 0.86 in year 1 and 0.39 in year 2), and Mn and Zn, although differences were only obvious for year 1 (0.61 vs. −0.08 in year 1 and 0.53 in year 2). These differences might be due to the measurement of elements in different tissues, as the ion balance is partly tissue-specific (Baxter, 2009). Nevertheless, the discrepancies with regard to the correlation between Mn and Zn need to be validated as they are only meaningful when regarding the results of year 1 in the work of Ding et al. (2010). Moreover, Wu et al. (2008) also found a significant positive correlation between Mn and Zn (0.61 in our study vs. 0.25 in Wu et al., 2008) when measuring 11 minerals (9 of which were identical with those from our research) in leaves of 183 *B. rapa* ssp. *pekinensis* DH lines grown in an open field. The relationship between Mn and Zn that we observed might be due to a locus with pleiotropic effects on Mn and Zn on the C genome that was so far unknown in *B. napus*. However, it cannot be verified by the results of Wu et al. (2008) as *B. rapa* represents the A genome of *B. napus*.

Not only relationships among traits representing the shoot ionome were evaluated but also those between mineral concentrations and traits characterizing seedling development. The relationship between K and H2O as well as Na and H2O (Figure 3B) is in all probability partly caused by the osmotic function of the two elements. But the similar chemical properties of alkali metals in general will also greatly influence the correlation between the two. The correlation between Ca and CIR, SOY, and ROU (Figures 2, 3B) hints at the cell wall stabilizing properties of Ca (Demarty et al., 1984). On the other hand, there is a tight negative correlation between Ca, K, and Na with most of the seedling development traits (except for PRK, ASR, CIR, ROU, SOY, and, as described before, H2O). Many of these ions are leaf growth-related. It is therefore conceivable that strong growth causes a dilution of these ions.

The identified relationships between the shoot ionome and seedling development raised the question whether one trait category might be predicted by the other. Multiple linear regression models were used to assess mineral concentrations as predictors for seedling development traits. The mean adj. *R*^2^ for the entire set was 35.6%. Körber et al. (2012) fitted linear models to predict agronomic and seed quality traits through the same set of seedling development trait data used in here. In 217 lines of a winter trial (subdivided into two subgroups) and 188 lines of a spring trial (all from the *B. napus* diversity set), the mean adj. *R*^2^ was 13%. We can therefore conclude that mineral concentrations are more suitable as predictors for seedling development traits than seedling development traits as predictors for agronomic and seed quality traits. This also implies that ionome data from field trials have the potential to predict agronomic data, but this will require further research.

### 4.3. Identified marker-trait associations

Heritabilities were moderate for the two traits Fe (0.46) and P (0.43) and high for all other traits (between 0.74 for B and 0.86 for S, Table 1), which decreased the power to detect marker-trait associations for the former traits. This might explain why no significant associations were found for the traits Fe and P (Supplementary Figure S1). Broadley et al. (2008) described a high heritability of Ca and Mg in *B. oleracea*, which was confirmed also for *B. napus* by the results from our study (0.85 for Ca, 0.77 for Mg, Table 1).

Owing to the manifold networks, pathways and transporters that influence the presence of minerals in plants, mineral concentrations are complex traits which are likely to be affected by a number of loci with small effects—a phenomenon that has been described before for different traits like yield (Shi et al., 2009). We observed on chromosome C9 two significant SNPs for Ca and another two SNPs for Mg, next to one significant association for Na and Zn, respectively (Table 3, Figure 4). The detection of association peaks for Ca and Mg are in line with the earlier findings of significant QTLs on C9 for Ca and Mg in *B. oleracea* (Broadley et al., 2008), although further studies are required to determine whether any of the loci from our and the previous research are in close proximity to each other, or even identical. The most pronounced association peak in this study was found for Na on A9, comprising seven SNPs of which four were in significant LD (Table 3, Figure 4, Supplementary Table S1). To search for annotated genes close to the significantly associated SNPs, an interval of 700 kb upstream and downstream of each SNP position was chosen as it corresponds to the extent of significant LD in *B. napus* (Bus et al., 2011), assuming a linear transformation with a rate of 0.674 Mb/cM according to Bancroft et al. (2011). The gene *SOS1* that was found in the proximity (about 540 kb) to the association hotspot is known to be an Na^+^/H^+^ antiporter that controls the uptake and efflux of Na, a mechanism which is crucial for plants under Na-rich conditions (Fraile-Escanciano et al., 2010). *SOS1* was first identified in *A. thaliana* (Wu et al., 1996), and its orthologs in *Brassica* species are also known (Chakraborty et al., 2012; Ford et al., 2012). Our identification of a closely situated gene that plays a key role in Na tolerance underlines the importance of the hotspot, which includes the genetic region Na-A9, in *B. napus*. The region might be particularly interesting in the framework of breeding for Na tolerance. However, it requires further research whether the hotspot is due to genotypic variation in *SOS1*.

Associated with the candidate genes within 700 kb upstream or downstream the significant SNPs, we identified a number of enriched GO terms that are functionally associated with the traits under study (Supplementary Table S3). When considering enriched GO terms across all traits it becomes obvious that these are mainly associated with general biological, cellular, and metabolic processes as well as enzyme and transporter activity and many cellular components involved in photosynthesis (Supplementary Table S3, Supplementary Figure S2). According to the detection of *SOS1*, the smallest *P* value (2.65 × 10^−7^) in the category biological process for Na was found for the term “sodium ion transport.” Furthermore, the second smallest *P* value (6.46 × 10^−6^) in the category molecular function for Na was found for “sodium ion transmembrane transporter activity,” and the GO slim term “transporter activity” was overrepresented for Na (Supplementary Figure S2). These findings support the assumption that the association hotspot is involved in Na uptake and transport. Many enriched terms hint at the known biological processes the examined traits are typically involved in, such as many photosynthesis-related terms for Cu, Mg, and Mn; ions that are essential for photosynthesis (Marschner, 1995). Similarly, phototropism was significantly enriched (*P* value 3.37 × 10^−4^) for biological process in Ca. A relationship between Ca and phototropism is expected because Ca controls the auxin-induced elongation of cells, and it has been described before in maize seedlings (Gehring et al., 1990). Hence, the enriched GO terms provide insights into what might be the roles of genes linked to the significantly associated SNPs we found.

With the association of the SNP Bn-ctg7180014703649-p1976 with Cu, Mn, and Zn (Table 3, Figure 4), we identified a locus with effects on three different heavy metals. This observation and the tight correlations between the three elements (Figures 2, 3A) suggest a pleiotropic effect of this SNP. On the other hand, the significant association with the three traits may be caused by linkage between the underlying genes. This hypothesis is supported by the extent of LD over distances of about 1 cM in the germplasm set under study (Bus et al., 2011). From the detected associated genes (data not shown), it is currently not obvious which one might be the causal gene or genes underlying this association. Additional approaches like RNA-seq analysis will be needed to reveal them.

Using SNPs from arrays in AM may cause ascertainment bias which brings forward an oversampling of mutations at intermediate frequencies, these again cause amounts of LD that are lower than those present in completely randomly selected SNP sets (Ingvarsson and Street, 2011). How strongly ascertainment bias affects the power of AM, however, is hard to say because it depends on various factors such as whether low- or intermediate-frequency SNPs are assumed to have larger effects on the trait under consideration (Clark et al., 2005; Manolio et al., 2009).

The significant associations found in this study explained between 3.02 and 9.73% of the phenotypic variation in single marker analyses (Table 3), and jointly up to 22.89%. The fact that none of the associations in single marker analyses explained more than 10% of the variation underlines the complexity of the examined traits, but it might also be due to imbalanced allele frequencies (Wricke and Weber, 1986). By way of example, for Ca, the SNPs Bn-ctg7180014762670-p15710 and Bn-Scaffold000019-p2724372 explain a comparable proportion of the variance (5.22 and 3.09%, respectively Table 3), but the minor allele frequency of the former is much lower (0.09) than that of the latter (0.47). These two SNPs could be studied further in a biparental population where allele frequencies would be balanced, and the SNP that explains a higher variance could be identified. Phenotypic differences in mineral concentrations, however, are not only due to the associations reported here but most likely also to further small effects of many undetected loci (Figure 4, Supplementary Figure S1).

## Author contributions

Anja Bus analyzed the data. Niklas Körber contributed the data on seedling development. Isobel A. P. Parkin provided the 6K array data. Birgit Samans and Rod J. Snowdon carried out the candidate gene and GO term enrichment analysis on the pre-published *B. napus* reference sequence. Jinquan Li supported the AM analysis. Anja Bus and Benjamin Stich wrote the manuscript. Benjamin Stich designed and supervised the study.

### Conflict of interest statement

The authors declare that the research was conducted in the absence of any commercial or financial relationships that could be construed as a potential conflict of interest.
