# Supplementary material for: Species- and genome-wide dissection of the shoot ionome in Brassica napus and its relationship to seedling development
Source: Front Plant Sci. 2014 Sep 30;5:485. doi: 10.3389/fpls.2014.00485 (PMC4179769; doi:10.3389/fpls.2014.00485)
Supplement: Supplementary file 1 [file DataSheet1.PDF]

Frontiers in Plant Science

Research Article
8 August 2014

Supplementary Material: Species- and genome-wide dissection of the shoot ionome in *Brassica napus* and its relationship to seedling development

Anja Bus [1,2], Niklas Körber [1,2], Isobel A.P. Parkin [3], Birgit Samans [4], Rod J. Snowdon [4], Jinquan Li [1] and Benjamin Stich [1,*]

[1] *Max Planck Institute for Plant Breeding Research, Carl-von-Linné-Weg 10, 50829 Cologne, Germany*
[2] *Institute of Crop Science and Resource Conservation, Crop Genetics and Biotechnology Unit, University of Bonn, Katzenburgweg 5, 53115 Bonn, Germany*
[3] *Agriculture and Agri-Food Canada, 107 Science Place, Saskatoon, SK S7N OX2, Canada*
[4] *Department of Plant Breeding, Research Centre for Biosystems, Land Use and Nutrition, Justus Liebig University, Heinrich-Buff-Ring 26-32, 35392 Giessen, Germany*

Correspondence*:
Benjamin Stich
Max Planck Institute for Plant Breeding Research, Carl-von-Linné-Weg 10, 50829 Cologne, Germany, stich@mpipz.mpg.de

1 SUPPLEMENTARY TABLES AND FIGURES

Supplementary Table S1. Squared correlations of allele frequencies between single nucleotide polymorphisms (SNPs) significantly associated with mineral concentrations. For details on SNPs, see Table 1

SNP No.	2	3	4	5	6	7	8	9	10	11	12	13	14	15	16	17	18	19	20	21	22	23	24	25	26	27
SNP No. 1: Bn-ctg7180014762670-p15710	0.949	0.001	0.121	0.254	0.124	0.010	0.012	0.011	0.035	0.006	0.018	0.050	0.036	0.036	0.036	0.036	0.055	0.052	0.024	0.098	0.098	0.230	0.005	0.129	0.024	0.000
SNP No. 2: Bn-ctg7180014762670-p5913		0.001	0.131	0.265	0.140	0.008	0.014	0.015	0.044	0.010	0.021	0.063	0.042	0.042	0.042	0.041	0.069	0.052	0.031	0.116	0.116	0.273	0.004	0.146	0.042	0.001
SNP No. 3: Bn-Scaffold000019-p2724372			0.000	0.021	0.032	0.104	0.026	0.028	0.058	0.110	0.118	0.286	0.265	0.256	0.265	0.286	0.224	0.050	0.163	0.001	0.001	0.010	0.118	0.005	0.000	0.056
SNP No. 4: Bn-ctg7180014703649-p1976				0.388	0.266	0.122	0.018	0.016	0.131	0.006	0.015	0.031	0.035	0.035	0.035	0.042	0.036	0.185	0.078	0.007	0.007	0.014	0.048	0.441	0.000	0.000
SNP No. 5: Bn-ctg7180014761247-p14206					0.405	0.040	0.054	0.055	0.145	0.016	0.028	0.106	0.095	0.094	0.095	0.101	0.131	0.121	0.047	0.040	0.040	0.094	0.005	0.376	0.005	0.006
SNP No. 6: Bn-ctg7180014763826-p6024						0.021	0.037	0.036	0.225	0.023	0.042	0.129	0.123	0.127	0.123	0.125	0.152	0.088	0.000	0.030	0.030	0.039	0.000	0.296	0.001	0.001
SNP No. 7: Bn-Scaffold000037-p374106							0.014	0.015	0.007	0.064	0.058	0.094	0.112	0.111	0.112	0.107	0.062	0.104	0.175	0.000	0.000	0.000	0.174	0.059	0.015	0.003
SNP No. 8: Bn-ctg7180014743366-p2908								1.000	0.062	0.022	0.034	0.107	0.107	0.102	0.107	0.103	0.070	0.000	0.026	0.006	0.006	0.029	0.025	0.024	0.003	0.005
SNP No. 9: p6_1705_snp15									0.062	0.023	0.034	0.109	0.108	0.103	0.108	0.105	0.071	0.000	0.026	0.006	0.006	0.029	0.026	0.023	0.004	0.005
SNP No. 10: Bn-Scaffold000002-p2620032										0.055	0.076	0.216	0.209	0.205	0.209	0.207	0.143	0.008	0.022	0.013	0.013	0.008	0.009	0.109	0.014	0.004
SNP No. 11: Bn-ctg7180014744730-p4896											0.902	0.165	0.217	0.208	0.217	0.218	0.120	0.005	0.090	0.002	0.002	0.008	0.061	0.007	0.000	0.001
SNP No. 12: Bn-Scaffold000022-p443574												0.186	0.241	0.231	0.241	0.243	0.140	0.001	0.094	0.005	0.005	0.011	0.054	0.018	0.003	0.001
SNP No. 13: Bn-Scaffold000040-p1595095													0.700	0.693	0.700	0.690	0.291	0.001	0.103	0.016	0.016	0.054	0.124	0.080	0.015	0.012
SNP No. 14: Bn-Scaffold000135-p101094														0.992	1.000	1.000	0.278	0.002	0.106	0.017	0.017	0.040	0.118	0.084	0.005	0.016
SNP No. 15: Bn-Scaffold000135-p393158															0.992	0.992	0.269	0.001	0.104	0.017	0.017	0.039	0.116	0.083	0.005	0.016
SNP No. 16: Bn-Scaffold000145-p148088																1.000	0.278	0.002	0.106	0.017	0.017	0.040	0.118	0.084	0.005	0.016
SNP No. 17: Bn-Scaffold000145-p66952																	0.271	0.002	0.108	0.010	0.010	0.033	0.108	0.087	0.001	0.021
SNP No. 18: Bn-Scaffold000481-p8079																		0.007	0.085	0.027	0.027	0.033	0.117	0.083	0.021	0.006
SNP No. 19: Bn-ctg7180014733138-p6820																			0.130	0.001	0.001	0.008	0.074	0.172	0.000	0.000
SNP No. 20: Bn-Scaffold000016-p1218862																				0.003	0.003	0.009	0.119	0.049	0.001	0.014
SNP No. 21: Bn-ctg7180014733119-p5210																					1.000	0.216	0.094	0.003	0.138	0.018
SNP No. 22: Bn-ctg7180014733119-p5336																						0.216	0.094	0.003	0.138	0.018
SNP No. 23: Bn-ctg7180014739774-p5570																							0.023	0.015	0.162	0.034
SNP No. 24: Bn-ctg7180014753773-p3940																								0.017	0.051	0.000
SNP No. 25: Bn-ctg7180014761298-p8042																									0.001	0.000
SNP No. 26: Bn-ctg7180014776046-p1871																										0.037
SNP No. 27: Bn-Scaffold000002-p221862																										

Supplementary Table S2. *Please find this as an additional .xls file.*

Supplementary Table S3. *Please find this as an additional .xls file.*

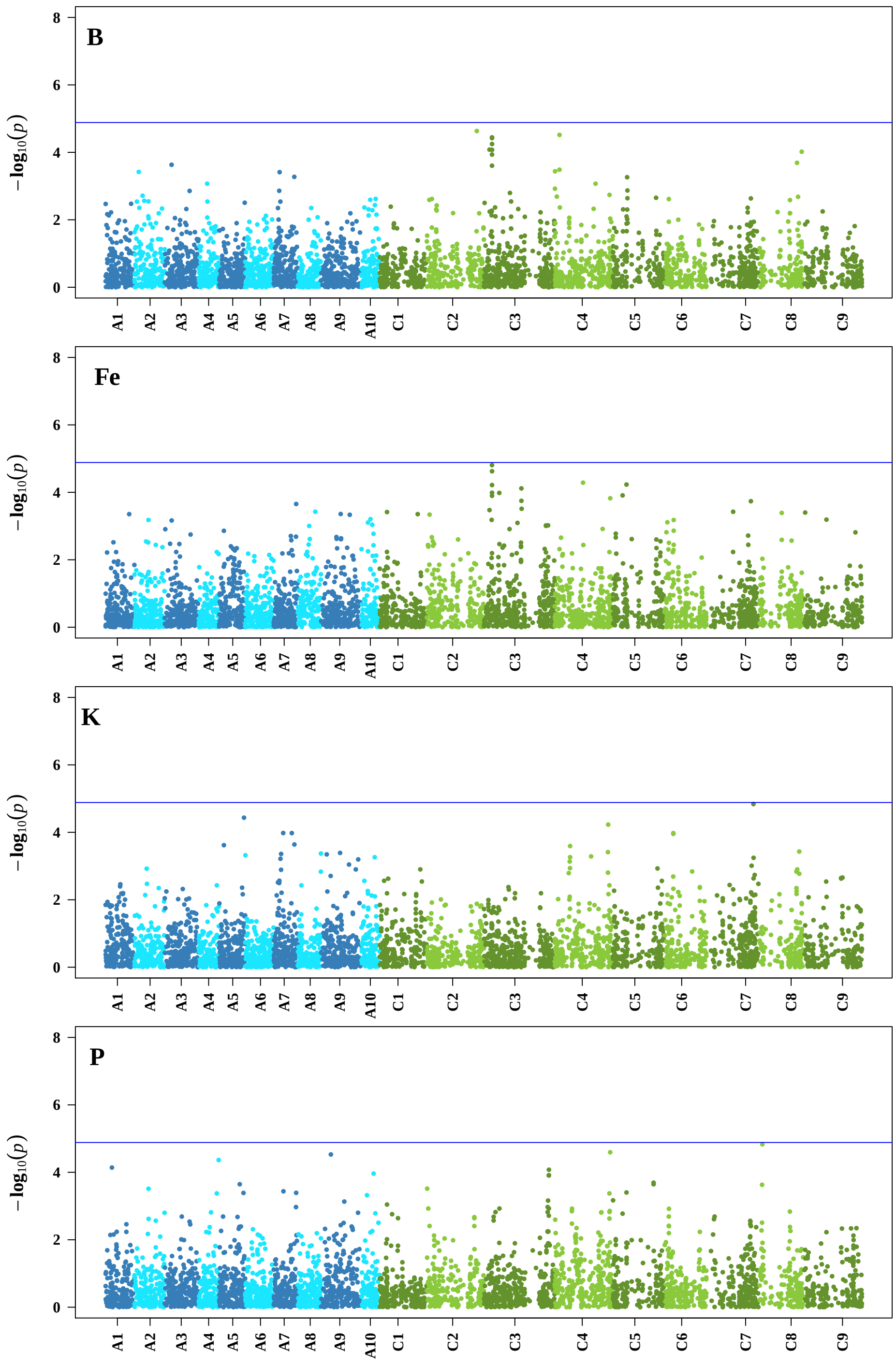

Supplementary Figure S1. Genome-wide *P* values for association analysis of four mineral concentrations for which no significant (α=0.05 prior to Bonferroni correction) associations were detected in a *B. napus* diversity set using a 6K single nucleotide polymorphism array after correction for multiple testing. Chromosomes of the *B. napus* A genome are colored blue, chomosomes of the *B. napus* C genome are colored green

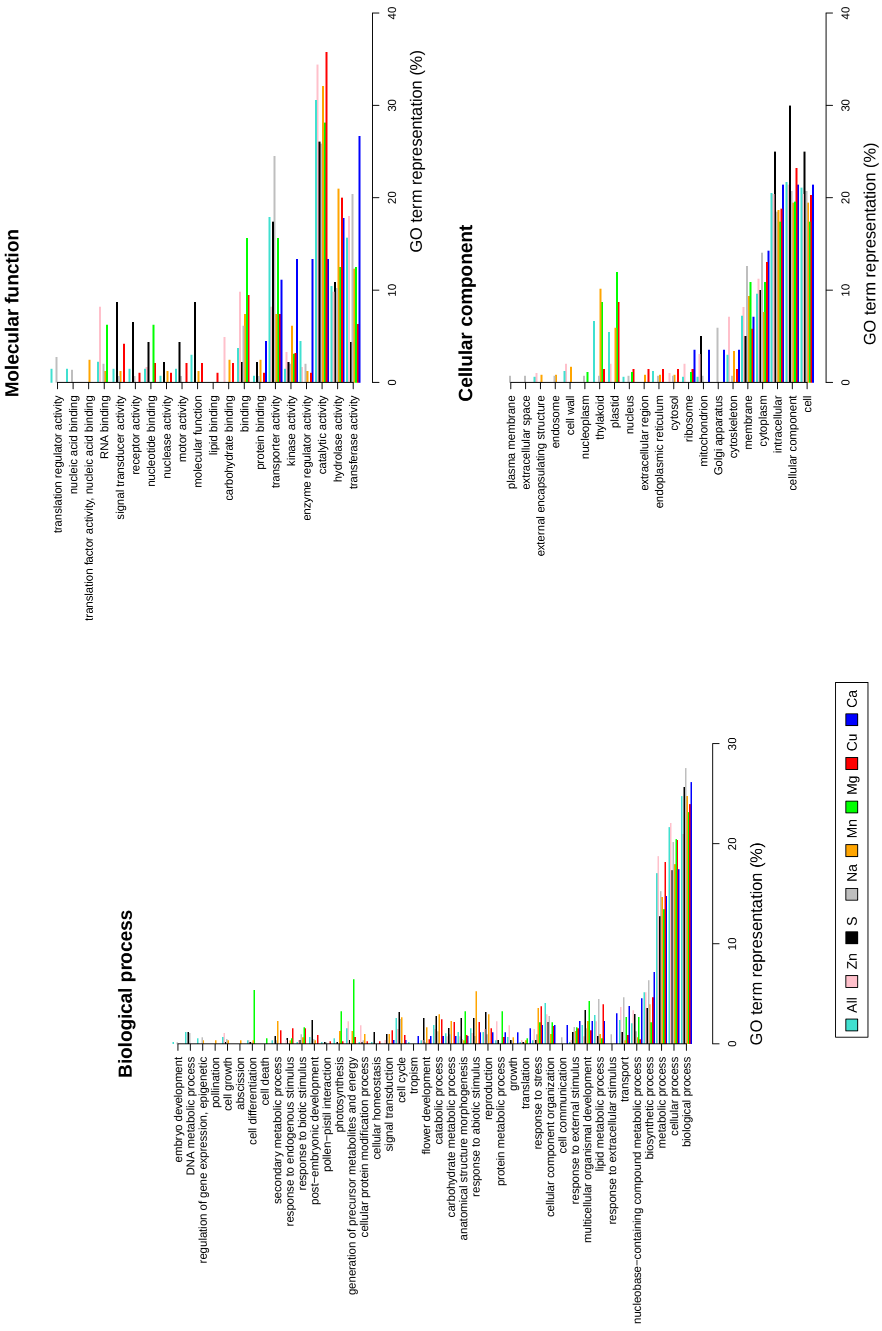

Supplementary Figure S2. Gene Ontology (GO) term representation (%) of enriched GO terms for eight traits describing the *B. napus* shoot ionome according to plant GO slim categories
